# ^18^F- based Quantification of the Osteogenic Potential of hMSCs

**DOI:** 10.3390/ijms21207692

**Published:** 2020-10-17

**Authors:** Tobias Grossner, Uwe Haberkorn, Tobias Gotterbarm

**Affiliations:** 1 Trauma Surgery and Paraplegiology, Clinic for Orthopedics and Trauma Surgery, Center for Orthopedics, University Hospital Heidelberg, 69120 Heidelberg, Germany; 2 Department of Nuclear Medicine, University Hospital Heidelberg, 69120 Heidelberg, Germany; uwe.haberkorn@med.uni-heidelberg.de; 3 Clinical Cooperation Unit Nuclear Medicine, DKFZ, 69120 Heidelberg, Germany; 4 Translational Lung Research Center Heidelberg (TLRC), German Center for Lung Research (DZL), 69120 Heidelberg, Germany; 5 Department of Orthopedics and Traumatology, Kepler University Hospital, Linz 4020, Austria; tobias.gotterbarm@kepleruniklinikum.at

**Keywords:** mesenchymal stem cells, osteogenic quantification, osteogenic differentiation, ^18^F, µ-PET, activimeter analysis

## Abstract

In bone tissue engineering, there is a constant need to design new methods for promoting in vitro osteogenic differentiation. Consequently, there is a strong demand for fast, effective and reliable methods to track and quantify osteogenesis in vitro. In this study, we used the radiopharmacon fluorine-18 (^18^F) to evaluate the amount of hydroxylapatite produced by mesenchymal stem cells (MSCs) in a monolayer cell culture in vitro. The hydroxylapatite bound tracer was evaluated using µ-positron emission tomography (µ-PET) scanning and activimeter analysis. It was therefore possible to determine the amount of synthesized mineral and thus to conclude the osteogenic potential of the cells. A Student’s *t*-test revealed a highly significant difference regarding tracer uptake between the osteogenic group and the corresponding control group (µ-PET *p* = 0.043; activimeter analysis *p* = 0.012). This tracer uptake showed a highly significant correlation with the gold standard of quantitative Alizarin Red staining (ARS) (r^2^ = 0.86) as well as with the absolute calcium content detected by inductively coupled plasma mass spectrometry (r^2^ = 0.81). The results showed that ^18^F labeling is a novel method to prove and quantify hydroxyapatite content in MSC monolayer cultures. The mineral layer remains intact for further analysis. This non-destructive in vitro method can be used to rapidly investigate bone tissue engineering strategies in terms of hydroxylapatite production, and could therefore accelerate the process of implementing new strategies in clinical practice.

## 1. Introduction

Critical-sized bone defects and non-unions are a challenging current problem in orthopedic and trauma surgery, not least because of an aging population [1]. The gold standard for treating substantial osteogenic defects is still autologous transplantation of spongiosa bone at the expense of donor site morbidity [2,3]. Multiple attempts have therefore been made in recent years to employ mesenchymal stem-cell-based therapy as a superior therapy option [4,5]. For this, mesenchymal stem cells (MSCs) are precultured into the osteogenic lineage in vitro before being applied in bone defects and non-unions. The huge advantage of these new therapies is the lack of donor site morbidity, which is a common complication when harvesting the autologous spongiosa (e.g., from the iliac crest or other sites) needed to fill the defect [4,5,6,7,8]. However, some data reflect lower clinical efficacy than initially assumed [9] which might, amongst other things, be due to ineffective differentiation.

In the field of bone tissue engineering, multiple procedures have been published to differentiate human bone-marrow MSCs and other stem cells into the osteogenic lineage. These use chemical or physical approaches to favor osteogenic differentiation [10,11,12,13]. It is hard to keep track of how efficiently these methods promote osteogenic differentiation without proper laboratory measurement methods. Having an efficient, sensitive, and reliable method to track and quantify the osteogenic differentiation potential of a protocol or cell type is the backbone of modern osteogenic tissue engineering. The strong need for new, non-destructive methods to image and quantify cellular response to osteogenic stimuli has already been stated [14]. A major problem with the common methods for osteogenic quantification is the destruction of cell cultures during the procedures, which means they are no longer available for further experiments [15]. Cell cultures must therefore be cultured in at least duplicates for extinctive analysis, which has a negative impact on both the costs and workload involved.

Radioactive tracers offer a superb opportunity for developing highly sensitive and non-destructive new methods for investigations in this specific field. A recent publication revealed that, if attached to a proper polyphosphonate, meta-stable technetium (^99m^Tc) is capable of binding with a high affinity to newly synthesized hydroxyapatite in two- and three-dimensional cell cultures. This uptake can be quantified using a gamma camera to determine the individual osteogenic potential [16,17]. In clinical medicine, this method has been known as bone scintigraphy since the 1970s [18]. As technetium is a gamma-radiation emitting radioactive source, the distribution of the tracer can be imaged three-dimensionally using single-photon emission computed tomography (SPECT). Downsides of this method are that it only allows low-resolution imaging and it has a lower sensitivity than other nuclear medicine methods [19]. Besides ^99m^Technetium, fluorine-18 (^18^F) is one of the most common tracers used to target hydroxyapatite in modern nuclear medicine. It was discovered as a bone scanning agent in the early 1960s for use in positron emission tomography (PET) [20]. After injection into the body, it is distributed via the circulatory system before it moves through the extracellular fluid space and binds to the crystal surface of bone [21]. After chemisorption onto hydroxyapatite, ^18^F exchanges rapidly for OH on the surface of the hydroxyapatite matrix (Ca_10_(PO_4_)_6_OH_2_) to form fluoroapatite (Ca_10_(PO_4_)_6_F_2_) [22]. Hence, ^18^F is a promising tracer for tracking hydroxyapatite in vitro, as it binds with a high affinity to the mineral and can be imaged with a µ-PET using small and very small probes [23]. ^18^F decays by emitting a pair of opposed gamma quants with 511 keV. This decay can be imaged with a high-resolution PET scanner, which is very sensitive to the emitted radiation and therefore very suitable for detecting even small amounts of tracer [23]. ^18^F has a very short half-life of just 110 min, which means that the radioactivity will decay to the background level within a short time of applying the tracer (after approximately five half-lives).

This study had two objectives: (1) to evaluate if ^18^F labeling works as a novel and non-destructive method to quantify the amount of extracellular hydroxyapatite deposition in a monolayer hMSC culture in an in vitro setting, and (2) to determine how the new method correlates with the standard hydroxyapatite quantification methods of Alizarin Red staining (ARS) and inductively coupled plasma mass spectrometry for validation.

## 2. Results

### 2.1. ^18^F µ-PET Analysis

**Figure 1:**
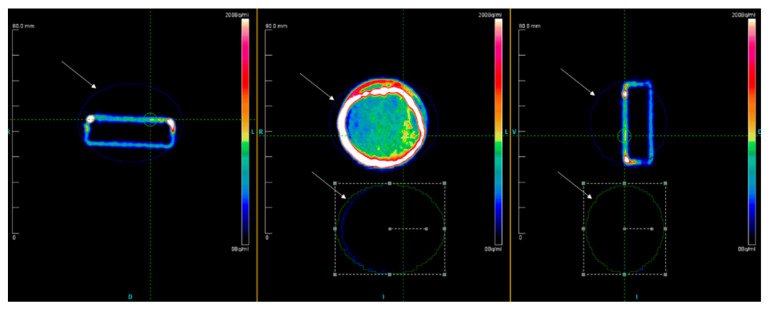
Exemplary µ-PET image of two dishes of one donor. Upper row of the dish was treated using osteogenic medium, while the lower row the dish was treated with control media. Blue and green outlines (indicated by white arrows) defining the regions of interest around the two dishes in axial, coronary and sagittal views.

There was substantial variability among human donors with respect to tracer uptake. Bound activity values in becquerel (Bq) showed a 20- to 60-fold higher tracer uptake in the osteogenic group (OSM) compared to the corresponding negative control group (CNTRL). As displayed in Figure 1, the µ-PET analysis revealed a 3D image of the distribution of the bound activity. The detected radioactive decay was calculated within the defined regions of interest (ROIs) (Figure 1).

The highest uptake within the six dishes treated with OSM was 49.3 Bq, while the lowest activity was still 6.1 Bq, reflecting the individual osteogenic potential of the donors. The mean activity within the OSM group was 18.15 Bq, with a standard deviation of 16.03 Bq and a standard error of the mean of 6.5 Bq. Within the OSM group, three donors showed a particularly higher uptake (49.3; 20.6; 13.4 Bq) compared to the three remaining donors (10.2; 9.3; 6.1 Bq). Nevertheless, even the donor with the lowest uptake within the OSM group (6.1 Bq) still had a 7.6-fold higher uptake than the donor with the highest uptake within the CNTRL group (0.8 Bq).

**Figure 2:**
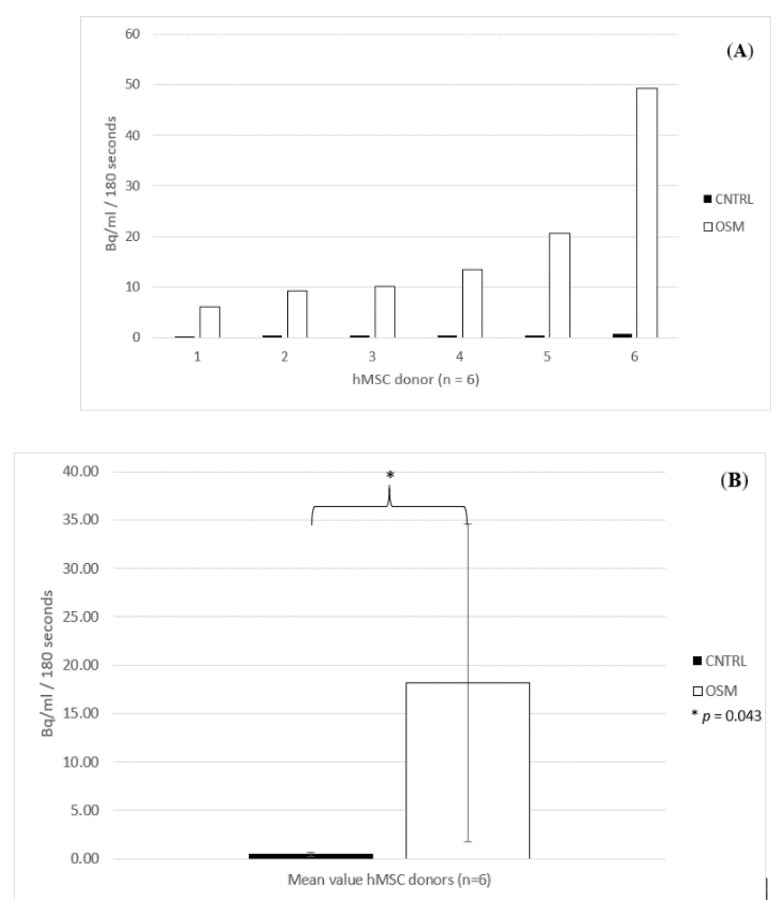
(**A**) Individual activity bound to each dish after 180 s of acquisition in µ-PET for donors 1–6 (*n* = 6) treated in osteogenic media (OSM) and the negative control group (CNTRL). (**B**) Mean activity bound to the dishes after 180 s of acquisition for the osteogenic media group (OSM) and the negative control group, +/− standard deviation, *p* = 0.043; * = significant difference between the two groups, *p* ≤ 0.05.

Within the CNTRL group, the highest activity measured for a dish was as low as 0.8 Bq, and the lowest activity was 0.29 Bq. The mean value for the CNTRL group was 0.46 Bq, with a standard deviation of 0.177 Bq and standard error of the mean of 0.072 Bq (Figure 2A,B).

### 2.2. Inductively Coupled Plasma Mass Spectrometry

**Figure 3:**
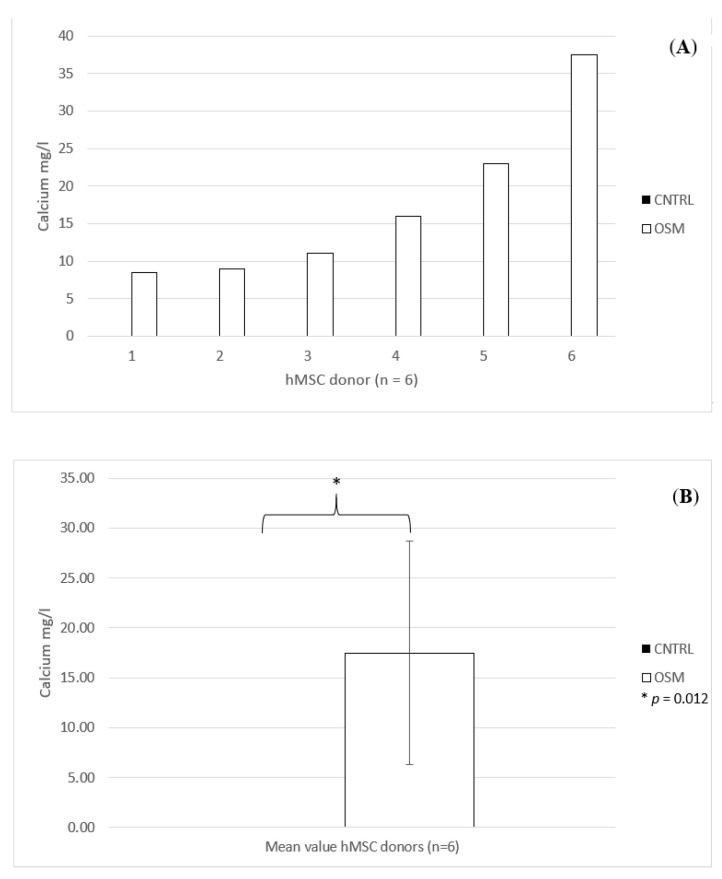
(**A**) Individual calcium content for each dish, donors 1–6 (*n* = 6) treated in osteogenic media (OSM) and the negative control group (CNTRL) determined by inductively coupled plasma mass spectrometry. (**B**) Mean values (*n* = 6) for the calcium content for the osteogenic media (OSM) and the negative control group (CNTRL), +/− standard deviation, *p* = 0.012; * = significant difference between the two groups, *p* ≤ 0.05.

Quantification of the calcium concentration of the monolayer cultures by inductively coupled plasma mass spectrometry (ICP-MS) likewise showed impressive results. Within the OSM group, the calcium concentration exhibited a certain variability (mean concentration 17.5 mg/L, standard deviation 11.20 mg/L, standard error of the mean 4.57 mg/L). The highest concentration was 37.5 mg/L and the lowest 8.5 mg/L. No relevant calcium concentration was detected in any of the CNTRL group samples: the concentration was 0 mg/L in all six samples (Figure 3A,B).

### 2.3. ^18^F Activimeter Analysis

**Figure 4:**
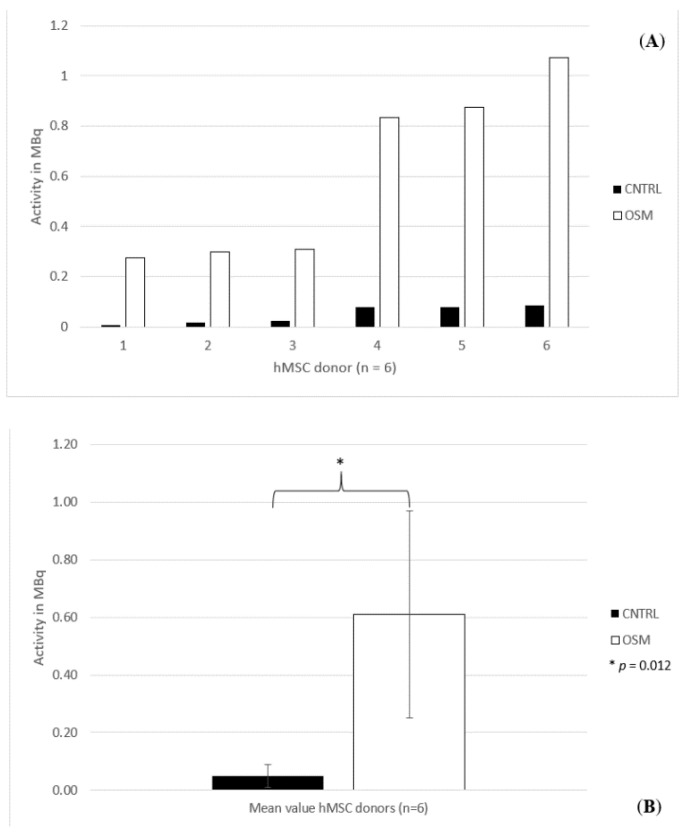
(**A**) Individual activity bound to each dish for donors 1–6 treated in osteogenic media (OSM) and the negative control group (CNTRL) measured by ^18^F-activimeter analysis. (**B**) Mean activity (*n* = 6) bound to the dishes in osteogenic media (OSM) and the negative control group (CNTRL) measured by ^18^F-activimeter analysis, +/− standard deviation, *p* = 0.012; * = significant difference between the two groups, *p* ≤ 0.05.

The analysis of the bound activity to the dishes within the OSM group showed a mean decay of 0.61 MBq (standard deviation 0.35 MBq, standard error of the mean 0.4 MBq) with a maximum of 1.07 MBq and a minimum decay of 0.275 MBq. The lowest decay in the OSM group was still three times higher than the highest uptake within the CNTRL group. In that group, the mean decay of the tracer was 0.049 MBq, with a maximum of 0.087 MBq and a minimum of 0.007 MBq (standard deviation 0.036 MBq, standard error of the mean 0.014 MBq) (Figure 4A,B).

### 2.4. Quantitative Alizarin Red Staining (ARS)

**Figure 5:**
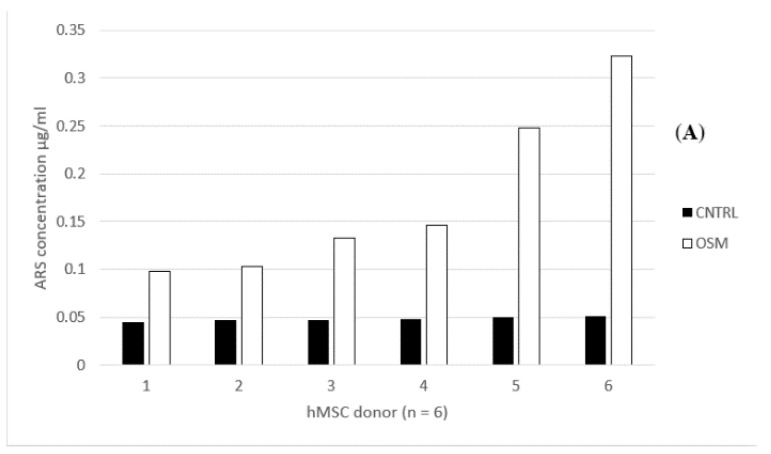

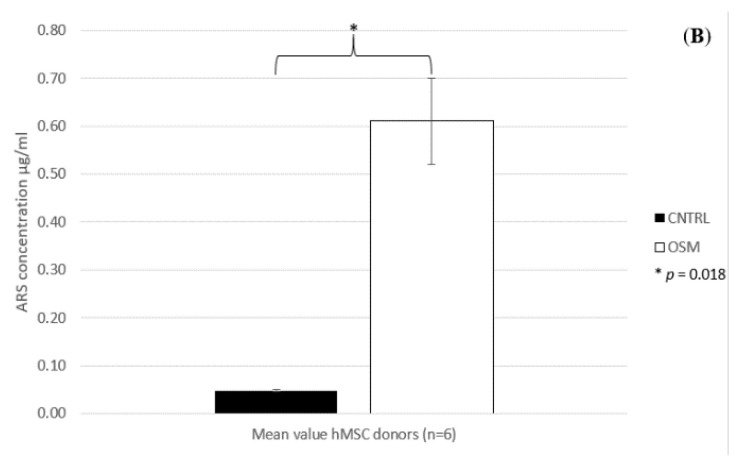
(**A**) Individual Alizarin Red stain (ARS) concentration in µg/mL for each dish, donors 1–6 treated in osteogenic media (OSM) and the negative control group (CNTRL). (**B**) Mean Alizarin Red stain concentration in µg/mL for the osteogenic media (OSM) and negative control group (CNTRL), +/− standard deviation, *p* = 0.018; * = significant difference between the two groups, *p* ≤ 0.05.

Within the OSM group, the mean ARS concentration was 1.24 µg/mL (standard deviation 0.819 µg/mL, standard error of the mean 0.334 µg/mL). The highest concentration within that group was 2.58 µg/mL, while the lowest concentration was 0.54 µg/mL. In contrast, within the CNTRL group, the highest uptake was 0.12 µg/mL while the lowest concentration was 0.06 µg/mL. The mean concentration was 0.09 µg/mL (standard deviation 0.021 µg/mL, standard error of the mean 0.008 µg/mL) (Figure 5A,B).

### 2.5. SEM and EDX Analyses

**Figure 6:**
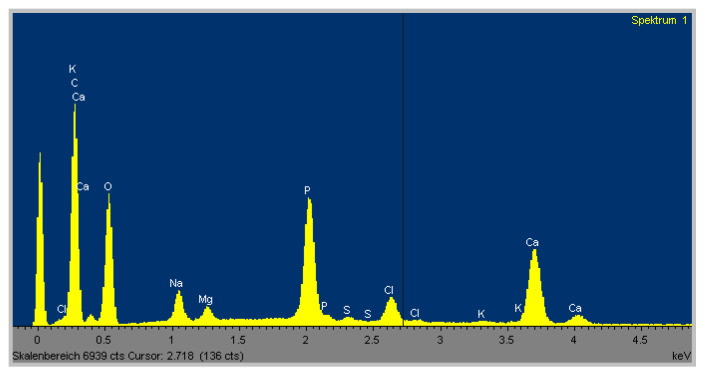
Energy dispersive X-ray microanalysis (EDX) spectrum of an exemplary dish from the osteogenic group showing the distinct presence of calcium and phosphorus. The various calcium peaks are caused by the proof of calcium at different energy levels. The high carbon peak is due to the carbon sputtering as demanded for proper EDX scanning.

**Figure 7:**
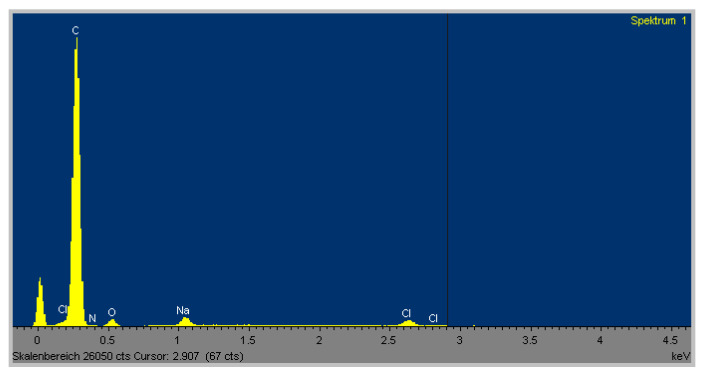
Energy dispersive X-ray microanalysis (EDX) spectrum of an exemplary dish from the negative control group showing no presence of calcium or phosphorus. The high carbon peak is due to the carbon sputtering as demanded for proper EDX scanning.

The EDX analysis of the elements revealed calcium was not present within the CNTRL group, although traces of phosphorus were detected. However, within the OSM group, there was a strong signal for the presence of calcium as well as for phosphorus. This corresponded with the SEM images, which showed multiple spots of a crystalline structure at 500× magnification within the OSM group. At the same magnification, no comparable structure was found within the CNTRL group (Figure 6 and Figure 7).

### 2.6. Statistical Results

A Student’s *t*-test was performed to detect significant differences regarding the uptake kinetics between the OSM and CNTRL groups for the ^18^F µ-PET analysis, inductively coupled plasma mass spectrometry, ^18^F activimeter analysis, and the quantitative ARS.

For the ^18^F µ-PET analysis, the Student’s *t*-test showed a significant difference between the two groups (*p* = 0.043).

Also, for the inductively coupled plasma mass spectrometry, the Student’s *t*-test revealed a significant difference between the two groups (*p* = 0.012). The same was true for the ^18^F activimeter analysis (*p* = 0.012).

Statistical testing of the ARS concentration within the two groups also revealed a significant difference between the two groups (*p* = 0.018).

To validate the new method (^18^F µ-PET analysis and ^18^F activimeter analysis), the results of the ^18^F µ-PET analysis were correlated with the results from the inductively coupled plasma mass spectrometry (ICP-MS) using a Spearman’s Rho correlation analysis. This revealed a highly significant correlation (*p* = 0.01) with a robust correlation coefficient (r^2^ = 0.819).

For validation of the ^18^F activimeter analysis, the results were correlated with the results from the quantitative ARS using the Spearman’s Rho correlation analysis. The results showed a highly significant correlation (*p* < 0.001) with a very robust correlation coefficient (r^2^ = 0.865).

Finally, a Spearman’s Rho correlation analysis showed a very high significant correlation (*p* < 0.001) with a particularly high correlation coefficient (r^2^ = 0.907) for the uptake measured by ^18^F activimeter analysis and the ^18^F µ-PET analysis.

## 3. Discussion

In this study, we evaluated the properties of ^18^F, a radioactive tracer that binds with a high affinity to newly synthesized hydroxyapatite. The exact amount of bound tracer (tracer uptake) can be determined using ^18^F µ-PET analysis as well as ^18^F activimeter analysis. After reviewing the results, we can present a novel, specific, non-destructive, and very accurate high-resolution imaging method to quantify the osteogenic potential of the cells, and respectively the osteogenic potential of the human donor, by assessing the amount of hydroxyapatite synthesized by osteogenic-differentiated human mesenchymal stem cells in monolayer cultures. One of the most important advantages of the new method is that the cell cultures remain intact after the ^18^F labeling for further analysis and experiments. This is verified by the results of the ARS procedure and the inductively coupled plasma mass spectrometry, as these procedures were performed using the cell cultures previously used for the ^18^F labeling. Therefore, no duplicate samples were needed to perform multiple quantitative analysis of the osteogenic differentiation.

While many modern evaluation and quantification methods evaluate osteogenic potential by determining the expression of specific proteins and enzymes, in clinical medicine it is most important to estimate the exact amount of mineral deposition (hydroxyapatite) because this is the most essential component for osteogenic regeneration [6,24]. As bone tissue engineering is a fast-growing field in translational medicine, new methods are continuously being implemented, and existing methods are improved to enhance the hydroxyapatite amount in vitro. Fast and reliable evaluations of these methods are important [12,13,25]. This need has already been recognized by other research groups who are performing investigations in the same direction by using fluorescence imaging for non-destructive imaging [26].

^18^F is a radiopharmaceutical known since the 1960s for its highly sensitive binding capacity to attach to newly produced hydroxyapatite by chemisorption. For the development of the new method, this key feature was utilized, as the tracer was directly applied to the monolayer cell cultures where ^18^F exchanges rapidly for OH on the surface of the hydroxyapatite matrix (Ca_10_(PO_4_)_6_OH_2_) to form fluoroapatite (Ca_10_(PO_4_)_6_F_2_) [21]. This chemical phenomenon, called uptake, was precisely quantified using ^18^F µ-PET scanning as well as ^18^F activimeter analysis. By using proper equipment like PET scanners, the exact uptake can be detected in terms of radioactive decay. The use of ^18^F for in vivo tracking of bone regeneration in rats using a µ-CT and PET was described earlier [27,28].

The advantage of the µ-PET method is that it can be used to perform high-resolution, three-dimensional imaging of the bound radioactivity within a cell culture dish while simultaneously quantifying the amount of bound tracer in terms of radioactive decay emitted from the tracer in becquerel [29]. By defining ROIs around specific areas of the cell culture, it is possible to determine the exact amount of radioactive activity there. Nevertheless, there is a minor loss in accuracy as not all emitted radiation will be detected by the partly open µ-PET system. Therefore, the most accurate determination of bound activity can be assessed by analyzing the probe in an activimeter. These devices have a shield probe tube into which the samples are inserted. Inside these probe tubes, almost the entire decay, reflected by emitted gamma radiation, is determined [29].

Our results show that the amount of tracer taken up is proportional to the amount of hydroxyapatite bound in each dish. At the same time, the amount of synthesized hydroxyapatite reflects the osteogenic potential of the individual donor. Within the OSM group, the results from the ^18^F µ-PET showed a 20- to 60-fold higher tracer uptake compared to the CNTRL group, while the spread of the uptake was between 49.3 and 6.0 Bq. However, within the control group the highest uptake was only 0.8 Bq. A possible explanation for the relatively wide spread of uptake values within the OSM group could be the individual osteogenic potential of the different human donors (n = 6) reflected by the individual mineralization potential. This emphasizes the strong need for an easily reproducible assay to assess the individual osteogenic differentiation potential for each individual (e.g., before clinically defining the best treatment strategy for non-union treatment) [30].

This phenomenon was also observed within the uptake results measured with the ^18^F activimeter analysis. Here, the spread of the uptake was between 1.07 and 0.275 MBq. Obviously, the unit used in ^18^F-µ-PET analysis was Bq while the decay in the activimeter was measured in MBq. This was caused by the circumstance that inside the activimeter almost all emitted energy is registered by the probe tube while in a µ-PET a partial volume effect occurs due to a limited resolution so that just a fraction of the total activity is measured, although the acquisition time of the ^18^F activimeter analysis is much shorter.

The Spearman’s Rho correlation analysis of the two methods showed a very highly significant correlation (*p* < 0.001), with a particularly high correlation coefficient (r^2^ = 0.907). Even if both methods quantify the emitted energy at 0.6335 MeV, it was anticipated that the correlation coefficient would be < 1, as the ^18^F-activimeter analysis has superior accuracy at the expense of the impracticality of imaging the samples, which is only possible with ^18^F µ-PET analysis.

The ^18^F µ-PET analysis was verified by correlating the results with the results of the absolute calcium concentration within the probes, which was assessed by inductively coupled plasma mass spectrometry. None of the specimens in the CNTRL group, analyzed by the highly sensitive mass spectrometry, showed any presence of calcium at all. This observation was ratified by the results from the energy dispersive X-ray microanalysis (EDX). Here, no calcium was detected within the sample of that group either. Therefore, it can be assume that, de facto, no mineralization process has occurred in the CNTRL group, while within the OSM group a distinct presence of the mineral was produced. The results of the ^18^F activimeter analysis were correlated with the results from the quantitative ARS method, which also reflects the amount of bound calcium within the probes. Spearman’s Rho correlation analysis showed a highly significant correlation (*p* = 0.01) while the correlation coefficient for the ^18^F µ-PET analysis with inductively coupled plasma mass spectrometry was r^2^ = 0.819 and for the ^18^F activimeter analysis with the quantitative ARS was r^2^ = 0.865. It can therefore be postulated that both novel methods were validated with a standard method. The proof of small amounts of ARS bound by the specimens of the CNTRL group probably occurred due to non-specific background staining.

Scanning electron microscopy (SEM) with energy dispersive X-ray microanalysis (EDX) of the OSM cultures revealed a precise signal for calcium and phosphorus while within the CNTRL group no calcium was detected. Only minimal traces of phosphorous were measured, which is probably a residue of the phosphate-buffered saline (PBS) washing performed in advance of the analysis.

It should be borne in mind that radioactive tracers are used in this method. These tracers emit a certain radiation that decays with a specific half-life. The decay is measured as activity in Bq or MBq. ^18^F has a comparably short half-life time of only 109.8 min, is produced in a cyclotron, and decays by ß+ decay at 242 keV [23,31]. The emitted positrons have a maximum range of only 2.2 mm in water. On reaching its rest energy, it consolidates with an electron and emits two photons with 511 keV in opposite directions to each other [29]. This radiation is detected by PET (coincidence-registration) for imaging and quantification. For an assay of 12 dishes, a total of 120 MBq ^18^F are needed. By using a dosimeter and following the radioprotection guidelines, this method can be considered as non-harmful to the examiner. We assume that the ^18^F labeling method, whether performed as ^18^F µ-PET analysis or as ^18^F activimeter analysis, is also capable of accessing and quantifying the amount of hydroxyapatite in three-dimensional cell culture systems. A limitation of the presented method is the need for an approved laboratory that has permission to work with radioactive material. Fortunately, most major hospitals now have a nuclear medicine unit or department where this method could be performed.

Additional studies will be performed to prove this assumption that could enhance this novel method, as high-resolution imaging of osteogenic 3D-cultures may help to identify hot spots of calcification within different tissue-engineered scaffolds without the need to destroy them. Further studies are also needed to determine if similar results can be achieved with less radiation.

## 4. Materials and Methods

### 4.1. Experimental Design at a Glance

Human mesenchymal stem cells (hMSCs) were harvested from the femoral bone cavity of healthy donors (*n* = 6), with approval from the Ethics Committee board, Faculty of medicine, University Hospital Heidelberg, Heidelberg Germany (No. S-309/2007; 11.10.2007), followed by cell expansion and subsequent osteogenic differentiation in 35-mm flat bottom Petri dishes. The dishes were seeded in double duplicates, while half of the dishes were cultured using a media known to favor osteogenic differentiation (OSM). A corresponding non-osteogenic negative control group (CNTRL) was cultured in parallel (EXP see Section 4.2). The dishes were then incubated with 10 MBq of the radioactive tracer ^18^F-sodium fluoride (^18^F-NaF) followed by µ-PET scanning and ^18^F activimeter analysis. Results from the µ-PET were validated by evaluating the exact calcium content of the specimen with the aid of inductively coupled plasma mass spectrometry. Results investigated by ^18^F activimeter analysis were validated using quantitative ARS. One dish of the osteogenic and the non-osteogenic negative control group was examined by SEM/EDX to prove the presence of hydroxyapatite.

### 4.2. hMSC Harvest and Expansion

Bone marrow aspirates were obtained from the proximal femoral cavity of six healthy donors (*n* = 6) under general anesthesia during an elective surgical procedure for total hip arthroplasty after informed consent. During preparation of the proximal femoral bone cavity, 10 mL of bone marrow was collected into a 20 mL syringe (BD Bioscience, Heidelberg, Germany) containing 1000 IU of heparin (Braun, Melsungen, Germany). Individual samples were diluted 1:1 with PBS (Gibco Life Technologies, Carlsbad, CA, USA) and washed twice with PBS. The mononuclear cell fraction was isolated by Ficoll gradient centrifugation (Ficoll-Paque-PLUS; GE Healthcare, Solingen, Germany). Mononuclear cells were plated in T-150 polystyrene tissue culture flasks (Falcon, Thermo Fisher Scientific, Waltham, MA, USA) at a density of 5 × 10^5^cells/cm^2^ and cultured in a humidified 5% CO_2_ atmosphere at 37 °C in low-glucose Dulbecco’s modified Eagle’s medium (DMEM-LG, Gibco Life Technologies, Carlsbad, CA, USA) containing 10% heat-inactivated (56 °C, 30 min) fetal bovine serum (Gibco Life Technologies, Carlsbad, CA, USA) and 1% Penicillin/Streptomycin (Gibco Life Technologies, Carlsbad, CA, USA)). This media was defined as “expansion media (EXP)”. After 48 h, non-adherent cells were removed by washing with PBS (Gibco Life Technologies, Carlsbad, CA, USA) while the adherent cells were defined as bone marrow MSCs. EXP media was changed every two to three days. At 90% of confluence, cells were trypsinized. For further experiments, these P0 cells were frozen in liquid nitrogen in 0.5 mL aliquots containing 5 × 10^5^ cells in DMEM-LG (Gibco Life Technologies, Carlsbad, CA, USA) with 20% FBS (Gibco Life Technologies, Carlsbad, CA, USA) and 10% DMSO (Sigma-Aldrich, Taufkirchen, Germany).

### 4.3. Osteogenic Differentiation Assay

P0 human MSCs (*n* = 6) were thawed and seeded into T-150 flasks (Falcon, Thermo Fisher Scientific, Waltham, MA, USA) with 250,000 cells each and cultured for 10 days to obtain enough cells for the further experiments. During this cell expansion stage, EXP media were used with changes every two to three days, cultured in a humidified 5% CO_2_ atmosphere at 37 °C. After 10 days, 80–90% confluence was reached, and cells were trypsinized and resuspended. Cells from every donor (*n* = 6) were then seeded at a density of 15,000 cells/cm^2^ in double duplicates (total of four dishes per donor) into 35-mm flat bottom Petri dishes (Corning, Corning, NY, USA). Cells from donor 6 were also seeded into two extra dishes for later SEM/EDX analysis.

Half of the dishes (two) of each donor (*n* = 6) and one of the extra dishes were treated with OSM to differentiate the human MSCs into the osteogenic lineage using DMEM-LG (Gibco Life Technologies, Carlsbad, CA, USA) containing 10% fetal bovine serum (Gibco Life Technologies, Carlsbad, CA, USA), 1% Penicillin/Streptomycin (Gibco Life Technologies, Carlsbad, CA, USA), and the osteogenic supplements 100 nM dexamethasone (Sigma-Aldrich, Taufkirchen, Germany), 10 mM b-glycerol phosphate (Sigma-Aldrich, Taufkirchen, Germany), and 0.173 mM L-ascorbic acid-2-phosphate (FUJIFILM Wako Chemical Corporation Europe, Neuss, Germany) (modified from [32]).

The other half of the dishes (two) of each donor (*n* = 6) and one of the extra dishes were treated with the same basal cell culture media but without the osteogenic supplements. These cell cultures served as a corresponding non-osteogenic negative control group (CNTRL). Cells were treated for 21 days with media change every two to three days cultured in a humidified 5% CO_2_ atmosphere at 37 °C.

### 4.4. ^18^F Labeling

After 21 days of cell culture in OSM or EXP, the medium was removed from the cultures, and the 35 mm-diameter dishes were carefully washed twice with PBS (Gibco Life Technologies, Carlsbad, CA, USA). The entire activity of ^18^F was confirmed with a dose calibrator (Activimeter ISOMED 1010, Nuklear-Medizintechnik Dresden GmbH, Dresden, Germany). An aliquot of 10 MBq ^18^F-NaF in 1 mL of 0.9% NaCl was added to each dish of the osteogenic group (OSM, *n* = 12) and to the corresponding non-osteogenic negative control group (CNTRL, *n* = 12). The dishes were then incubated at room temperature for 2 h. The remaining liquid tracer was removed, and the dishes were washed three times (for approximately 30 min) in PBS (Gibco Life Technologies, Carlsbad, CA, USA) to remove the unbound radiotracer.

Two blank samples were treated the same way as the dishes containing the cell culture.

### 4.5. ^18^F-µ-PET Analysis

Subsequently, half of the dishes (OSM *n* = 6, CNTRL *n* = 6) were scanned for 180 s in a µ-PET (Positron Emission Tomograph, Siemens Healthcare, Erlangen, Germany) to produce a 3D image of the bound activity distribution and to determine the exact amount of bound radioactive activity. Xeleris Software (GE Healthcare, IL, USA) was used to post-process the images acquired. A standardized ROI, relative to the diameter and volume of the cell culture dishes, was defined around each individual dish. Then, the bound radioactive activity for each ROI was calculated (Figure 1) in becquerel (Bq).

The blank amount of activity was deducted from the measured amount of activity in each dish.

The dishes were then stored until the radiation decayed to background levels.

### 4.6. Inductively Coupled Plasma Mass Spectrometry

The cell cultures previously analyzed for ^18^F µ-PET were prepared for the absolute calcium quantification using inductively coupled plasma mass spectrometry by first of all lysing the monolayer cultures to remove the bound calcium. This was done by washing with PBS (Gibco Life Technologies, Carlsbad, CA, USA) twice and then adding 2 mL 2 M HCl (Sigma-Aldrich, Taufkirchen, Germany) to mobilize the bound calcium. After 12 h of incubation at room temperature, the solution was neutralized by adding 2 mL 2 M NaOH (Sigma-Aldrich, Taufkirchen, Germany). Subsequently, the lysate was analyzed using an inductively coupled plasma mass spectrometer (Perkin Elmer ELAN6100 ICP-MS. Rodgau, Germany).

### 4.7. ^18^F Activimeter Analysis

The other half of the ^18^F labeled dishes (OSM *n* = 6, CNTRL *n* = 6) were analyzed for just the bound radioactive activity (in MBq) without visualization using an activimeter (ISOMED 1010, Nuklear-Medizintechnik Dresden GmbH, Dresden, Germany). The detection window was set for ^18^F decay (0.6335 MeV), and the entire dish was put into the analyzation slot of the activimeter to acquire the amount of decay.

The dishes were then appropriately stored until the radiation decayed to background levels.

The blank amount of activity was deducted from the measured amount of activity in each dish.

### 4.8. ARS

For additional hydroxyapatite quantification of the cell cultures, the same dishes were used as for the previously earlier investigated ^18^F activimeter analysis.

One dish from each group (OSM, CNTRL) and donor (*n* = 6) was used. The 35-mm dishes were fixed with 2.5 mL 70% ethanol (−20 °C) for 4 min followed by washing with aqua dest. The staining solution was prepared by dissolving 0.5 g Alizarin Red S powder (Sigma-Aldrich A5533, Taufkirchen, Germany) in 100 mL aqua dest. The pH was adjusted by adding ammonium hydroxide (Sigma-Aldrich, Taufkirchen, Germany) until a pH of 7.2 was obtained, then 1 mL of staining solution was added to each dish. The dishes were incubated at room temperature for 10 min followed by repeated (5×) washing with 1 mL aqua dest. Next, 1 mL of a 10% (*w/v*) cetylpyridiniumchloride (Sigma-Aldrich, Saint Louis, MO, USA) solution dissolved in 10 mM sodium phosphate (Sigma-Aldrich, Taufkirchen, Germany) was added to each dish. After a new incubation for 10 min at room temperature on a shaker, 20 µL from each sample was placed in a 96-well plate and 180 µL of the 10% (*w/v*) cetylpyridiniumchloride (Sigma-Aldrich, Saint Louis, MO, USA) solution was added. In parallel, a standard dilution with Alizarin Red concentrations between 62.5–0.01 µg/mL was prepared in the same 96-well plate. Photometric absorption was determined at 570 nm in a 96-well plate reader, followed by plotting a standard curve with the results of the standard dilution. The absorption values of the samples were transferred into Alizarin Red concentration by referring to the standard curve.

### 4.9. SEM and EDX Analysis of MSC Cultures

The two extra cultured dishes with hMSC cultures (one with OSM media, one with EXP media) were carbon sputtered and investigated using a LEO 440 scanning electron microscope SEM (LEO 440 REM, Zeiss, Oberkochen, Germany). The backscattered mode was used for element-weighted images, and element analysis was performed using the Inca EDX-System (Oxford Instruments, Oxfordshire, UK).

### 4.10. Statistics

A Student’s *t*-test was performed to determine the significant difference regarding the bound activity in Bq, the calcium concentration and the Alizarin Red concentration between the OSM and CNTRL groups.

Prior to this test, the results were tested for normal distribution using the Kolmogorov–Smirnov test.

Statistical analyses were performed using SPSS (IBM Deutschland GmbH, Ehningen, Germany) Statistics Version 20. Statistical significance was set to *p* < 0.05.

## Abbreviations

ARS	Alizarin Red staining
Bq	Becquerel
CNTRL	Non-oste ogenic negative control group
EDX	Energy Dispersive X-ray Microanalysis
hMSC	Human mesenchymal stem cells
ICP-MS	Inductively coupled plasma mass spectrometry
MeV	Mega-electron volts
MSC	Mesenchymal stem cells
OSM	Osteogenic treatment group
PET	Positron emission tomography
ROI	Region of interest
SEM	Scanning Electron Microscopy
SPECT	Single-photon emission computed tomography
